# Strategies for seeking care in the host country among asylum-seeking women who have been victims of sexual violence: A French qualitative study

**DOI:** 10.1016/j.jmh.2024.100254

**Published:** 2024-07-27

**Authors:** Khouani Jeremy, Anne Desrues, Constance Decloitre-Amiard, Marion Landrin, Rachel Cohen Boulakia, Didier Thery, Gaëtan Gentile, Pascal Auquier, Maeva Jego

**Affiliations:** a Department of General Practice, Faculty of Medicine, Aix-Marseille University, 27 Bd Jean Moulin, Marseille CEDEX 5 13385, France; b UR3279 CERESS, Aix-Marseille University, Marseille, France; c Department of Public Health, University Hospital APHM, Marseille, France; d INS UMR1106, Aix-Marseille University, Institut des Neurosciences des Systèmes, France

**Keywords:** Sexual violence, Asylum seeker, Qualitative study, Healthcare system

## Abstract

**Background:**

Incidence of sexual violence among recently arrived asylum-seeking women in France (INCIDAVI) is a French study which found a past year incidence of 26 % for postarrival sexual violence (SV) among asylum-seeking women (ASW). It reported that fewer than 1 out of 10 victims consulted a healthcare professional when SV occurred. These findings raise the question of how ASW who have been victims of SV get involved in care. We aimed to explore the mechanisms and obstacles to seeking care in the host country among this population.

**Methods:**

This qualitative phase of INCIDAVI was based on a grounded theory approach. Twenty semi structured interviews were conducted between February 1, 2022, and July 29, 2022. The interviews explored the conditions under which women talk about SV, the care pathway in France and the perceived consequences of care. We performed an inductive analysis using NVivo® 14 software.

**Findings:**

Life paths of ASW are traversed by SV which influence their health and safety behaviour and can re-expose them to SV. Talking about SV is a rare choice focused on seeking protection. When appropriate care is used, it is perceived as beneficial and leads to a change in the perception of a possible recovery.

**Interpretation:**

The failure of ASW to seek care for SV is shaped by the fact that SV is initially perceived as ordinary. A proactive attitude on the part of carers towards detecting such violence leads to positive experiences of care, which in turn influence women's initial perceptions of SV, enabling them to envisage health recovery.

## Introduction

Nearly 1 out of 3 women in the world has experienced lifetime physical or sexual violence (SV) from an intimate partner, (World Health Organization (WHO), 2017; Sardinha et al., 2022) and 6 % have experienced nonpartner SV (https://www.who.int/publications/i/item/9789240022256). Among women, the World Health Organization (WHO) reported that those who are forcibly displaced to escape war or persecutions are particularly vulnerable to SV (World Health Organisation (WHO), 2018). The WHO also recommended distinguishing the administrative status of these forcibly displaced persons (undocumented migrants, refugees, asylum seekers) when designing care and research programs. Asylum seekers are displaced persons who have recently arrived in their host country and whose administrative situation is under examination. These unstable social conditions (related to residence permits, health insurance, housing) and cultural barriers hinder the use of healthcare (Cignacco et al., 2018; Marek et al., 2019). In 2022, 881 220 first-time asylum seekers applied for international protection in Europe, 29·2 % of whom were women (https://ec.europa.eu/eurostat/statistics-explained/index.php?title=Annual_asylum_statistics). In France, 41 540 women registered for a first asylum application in 2022 (Office, 2022)

Asylum-seeking women (ASW) are highly exposed to SV: a metanalysis published in 2023 found a lifetime prevalence of SV at 44 % among ASW (Cayreyre et al., 2024). The consequences of SV on the health of ASW, including depression (27 %), posttraumatic stress disorder (45·1 %) (Nesterko et al., 2021), and severe pelvic pain (51·6 %), have also been described (Masterson et al., 2014). Therefore, the United Nations High Commissioner for Refugees (UNHCR) identified the prevention and detection of SV and care for ASW who have experienced SV as a priority for host countries (https://www.unhcr.org/publications/operations/3b9cc26c4/sexual-violence-against-refugees-guidelines-prevention-response-unhcr.html, https://www.unhcr.org/fr-fr/publications/operations/4ad2f840e/violence-sexuelle-sexiste-contre-refugies-rapatries-personnes-deplacees.html).

INCIDAVI (Incidence of sexual violence among recently arrived asylum-seeking women in France) is a retrospective cohort study conducted on 273 ASW who had lived in France for more than one year but less than two. The study found a past-year incidence of 26·3 % for postarrival SV and 4·8 % for postarrival rape (a rate that is 18 times higher than that for the general population of the host country (Khouani et al., 2023)). It also reported that more than half of women who were victims of SV had not sought any assistance when SV occurred and that fewer than 1 out of 10 consulted a healthcare professional. These findings raise the question of what the appropriate health care is for these women in their host countries. In particular, we need to know more about how ASW who are victims of SV relate to health and care to improve SV detection and medical care for this vulnerable population. Previous European studies have investigated SV suffered by ASW (De Schrijver et al., 2022; Keygnaert et al., 2012; Keygnaert and Guieu, 2015; Oliveira et al., 2018). Reducing social isolation and improving sexual health education would seem to improve prevention of SV and care for female victims in this population. All these studies highlighted the need to improve knowledge in this area. Exploring the experiences of ASW suffering SV would give us a better understanding of how we can address unmet health needs and lack of social support. This could also make it possible to identify ways of increasing the rate of care-seeking in cases of sexual violence, which was so low in our first quantitative section of INCIDAVI.

The aim of this qualitative study was to explore the mechanisms and obstacles to seeking care in the host country among ASW who had been victims of SV prior to or after their arrival in France. It corresponds to the qualitative part of our survey on SV suffered by ASW in host countries (Khouani et al., 2023).

## Methods

### Study design

We conducted a qualitative study based on grounded theory approach from April to June 2022 with ASW who had been victims of SV.

### Population and sampling

The source population for our study was the women included in the INCIDAVI survey; the construction of the cohort (e.g., inclusion and exclusion criteria) and the way to detect SV is described in a previous report of the first part of our project (Khouani et al., 2023). For this qualitative phase, we used a purposeful variation sampling method and progressively selected women from INCIDAVI for inclusion in a targeted way to obtain a diversified sample with respect to age, language status, geographical origin and SV status (prior to or after arrival in France). Purposive sampling based on pre-identified variables that may influence individuals' experiences is the most commonly used sampling method in qualitative research (Palinkas et al., 2015, Marshall, 1996). Sampling was carried out as the interviews progressed in order to test the hypotheses put forward against new profiles. We did not define a minimum sample size and we stopped including participants in the sample when data saturation had been reached (defined as two successive interviews no longer offering any new data or hypothesis or the need to continue diversifying the sample). Twenty women were interviewed between February 1, 2022, and July 29, 2022. Data saturation was reached at the 18th interview and confirmed by the 19th and 20th interviews. The average duration of the interviews was 1 h (with a range from 15 min to 75 min). Sixteen interviews were conducted at a partner hospital, and 4 were conducted by phone. A professional interpreter was necessary for 1 interview.

### Data collection

Two trained English- and French-speaking researchers conducted face-to-face semistructured interviews at a partner hospital in a private place or by phone when participants did not wish to travel. The interviewers were experienced in the field of sexual violence, one a sociologist involved in an NGO for victims of incest and the other a general practitioner. Both had been specifically trained for 2 days in conducting semi-structured interviews by a researcher experienced in surveys on asylum and sexual violence. The interviews were audio-recorded and then fully transcribed. It was explained that this survey was designed to study the living conditions of asylum seekers in their host country and their possible exposure to sexual violence. Participants were reimbursed for their travel expenses. If an asylum-seeking female wanted to participate in the study but did not wish to travel, a telephone interview was offered instead. In case of a telephone interview, they had to take place with the participant concerned directly and only, who had to be in a confidential place. A professional telephone interpretation service was used for non-French-speaking and non-English-speaking participants. These professional interpreters were experienced in interviewing ASW. The interview guide explored through 8 questions the women's conditions for speaking out about SV and requesting help, the care pathway in France and perceived consequences of care. The state medical services responsible for the reception of asylum seekers in France were partners in the study and helped to draw up the guide. The understanding and acceptability of the interview guide was tested before conducting the study with two peer workers refugee women as part of a participatory research approach. As the interviews progressed, the guide was further adapted to clarify the emerging results and address the hypotheses. Beginning with the 5th interview, a sixth theme was integrated relating to the evolution of representations of SV and, more broadly, the protection of women. To improve objectivity, the researchers identified their emotions, the evolution of their assumptions, and their intellectual process for analysis in a logbook (Lejeune, 2014).

### Analyses

Three researchers (C.D. M. J and A.D.) triangulated the analysis, with regular discussions about the key stages of analysis with a fourth researcher (J.K.). In this inductive approach, the themes were not identified in advance but derived from the data. We first analysed the interviews one by one, coding the verbatim line by line (open coding). We assigned codes to groups of words, sentences or paragraphs, exploring the meaning of the interviewers' discourse (experiential coding). Secondly, we carried out axial coding, on a grid common to all the interviews, using the codes and their underlying data to find broader themes (properties). Thirdly, links between properties were identified, and properties were grouped and summarised into categories (Lebeau et al., 2021; Glaser and Strauss, 2009; Paillé and Mucchielli, 2021). At the end of the analysis process, the integration of our results with the literature led to a theorisation scheme. We used NVivo® 14 software for this analysis.

### Ethical issues

The study was explained to the participants, and oral consent was obtained before their inclusion in the study. All interviews were anonymized as soon as they were conducted. The study protocol applied the WHO ethical and safety guidelines for researching violence. This study received authorization from the ethical committee South-Mediterranean V under the authority of the French Ministry of Research (N° approval number 21.04.02.59049). As the age of majority in France is 18, the ethics committee asked us to limit our survey to women who had reached the age of majority. Written informed consent was obtained from all subjects.

### Role of the funding source

The study received funding from the Ministry of Health in the amount of 50,000 Euros, which was used to cover the costs of professional interpreters, human resources for data collection, and translation costs. The budget was collected and managed by the public research department of the public assistance hospitals of Marseille.

## Results

**Fig. 1:**
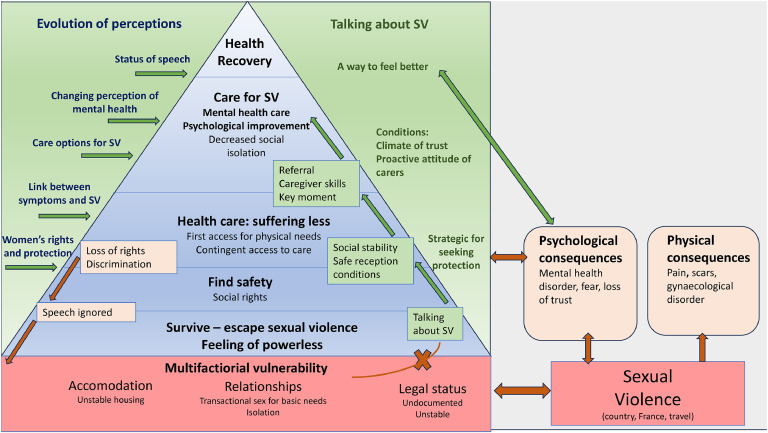
Theorization scheme: conditions for health recovery.

The results are presented in the following order: Characteristics of the women included, Coding tree, respective presentation of the 4 categories of analysis by order of emergence and finally the grounded theory with its related schema (Fig. 1).

### Characteristics of the women included

Most of the women (13 of 20) were from West Africa. None of them were from South America. They had been in France for at least one year but less than two years and all had health insurance. Eighteen had been victims of SV in France (including 4 women who had been raped), and 17 had been victims before arriving in France. Eleven had been victims of SV both prior to and after their arrival in France (Table 1).

**Table 1:** Women's demographic characteristics and history of SV (*n* = 20).

Characteristic	Number of women
Age	
< 30 years	5
30 to 40 years	13
> 40 years	2
Language	
French	14
English	4
Other	2
Geographical origin	
West Africa	13
Maghreb	1
Rest of Africa	2
Middle east	1
Europe	1
Asia	2
Number of dependent minor children in the past year	
0	5
1	8
>1	7
Couple relationship with partner present in France	
Yes	9
No	11
Main accommodation during the past year	
No support system	9
Single dwelling with legal lease	2
National Asylum Support System	6
Emergency housing support	3
Financial resources	
None	2
State financial aid	11
Undeclared work	3
Family, friend or associate support	2
Work with legal contract	2
Sexual violence prior to arrival in France	
Yes	17
No	3
Sexual violence in France	
Yes *Including rape*	18 4
No	2

#### Coding tree

We identified 285 experiential codes, which defined 20 properties, from which 4 categories emerged (Table 2): (1) Previous life circumstances related to sexual violence influence women's adverse health outcomes, risk behaviours, and new exposures to sexual violence, (2) Talking about SV: a rare and strategic choice focused on seeking protection, (3) A contingent use of care in cases of SV, but with a very beneficial impact, (4) A representation of seeking care in cases of SV, shaped by past experiences of care.

**Table 2:** Coding tree with main categories and properties.

Categories	Properties
(1) Previous life circumstances related to sexual violence influence women's adverse health outcomes, risk behaviours, and new exposures to sexual violence	• Strategies to escape SV • Life paths traversed by SV • Other priorities than caring • Consequences of SV • Repeted obstacles and discouragement • Justice: unthought and not now • Insecurity factors • Safety factors
(2) Talking about SV: a rare and strategic choice focused on seeking protection	• Utility of speech to find help • A limited, conditional and risky speech • The need to be soothed to be able to speech • Condition of the interlocutor and capacity fos listen to enable speech • With trusted or referred persons • Towards reconstruction (physical, psychological)
(3) A contingent use of care in cases of SV, but with a very beneficial impact	• Care process for SV • Strategies for seeking care
(4) A representation of seeking care in cases of SV, shaped by past experiences of care	• Differences with home countries on care and caregivers' attitudes • To be a self-sufficient and helpful woman • Changing perceptions of mental health as care becomes available • Views about specific care for SV consequences: excision, AIDS

### Previous life circumstances related to sexual violence influence women's adverse health outcomes, risk behaviours, and new exposures to sexual violence

The women we met in our study reported that their lives had been shaped by SV. The violence was described as tolerated or organized by the local authority figures in the birth country.

"*I lived through my forced marriage. They gave me a dowry. I left. The first night wasn't easy. He wanted to have sex with me by force. I didn't want to. He looked for my family. The second night, they came. Two people came to hold me; two held my hand, and two other people took my feet*" (interview 6).”

*“I think there's nothing to be done. That's what it was like. I'd say to myself, I accept the situation. In any case, there's nothing to be done. It's there, it's there, it's happened, there's nothing you can do about it.” (interview 1)*

France remained part of the "life course" of the women, with similar risks of violence and structural insecurity before possibly being the hoped-for place of stability.

*“On the second day, I also slept at the train station. […] They asked me, they told me. It's like he comes to flirt with me, but if I say no, he actually does it by force” (Interview 12)*

The factors they identified as making one vulnerable to SV were predominantly related to poor social conditions: housing instability, lack of money, food and loss of stability due to losing residence permit (for whom asylum had been refused). For the women, the first arrival in France was itself risky due to language barriers, isolation and misunderstanding of the system.

*“In terms of violences, when I came to France, the only violence that I had with [name], because by then I was sick. I got raped by him because I don't know anything, I don't know anybody. Even the asylum, I was thinking that it was a hospital place where we were going. I don't know anything by then. I was really…. I couldn't talk, I couldn't remember a lot.” (Interview 8)*

The precariousness of their housing situations forced the women to urgently and hastily look for active solutions, which could expose them to danger, for example, engaging in transactional relationships or experiencing aggression from the people who housed them or when they visited squats.

"*Yes, at the train station. Then, I found this man... who offered for me to come and stay with him... he has a flat in which he lives, just until we find a solution. So I accepted because I had no choice. I've got no choice. But then he raped me, several times. He hit me (*cries*). And if I said no, he'd tell me to "get out*". *So I stayed there because it was... it was November at the time. It's cold outside, I don't know anyone*. (Crying)" (interview 20)

In response to danger, the women developed individual defences and protection strategies. Fear and the desire to escape SV led the women to remain in one place when they found a safe place to stay. This strategy led to a loss of social ties that isolated them. Stabilizing accommodations from organizations such as associations or reception centres for asylum seekers were a source of well-being and a relative feeling of protection.

*“For the moment, as I am in the house, I am fine. I am protected, I am well”* (Interview 9)

Obtaining refugee status symbolized a way out of long-term vulnerability, as well as access to effective legal protection against SV. Applying for asylum was thus a time of anxious waiting and hope.

*"We can't risk complaining to the police. We can't risk going to the police. We're scared. I always thought that it was those with legal papers who were entitled to that. To make that request. To file a complaint”*. (Interview 4)

### Talking about SV: a rare and strategic choice focused on seeking protection

Although the women reported that they first sought help from friends and family after suffering SV, talking about the SV was risky and painful. The risks involved in recounting SV to friends and family included shame and rejection; their words could be reported and used against them.

"*In fact, it's something very intimate that we can't share with just anyone. There are people who are likely to go and talk about it, so it's something we keep to ourselves*." (interview 4)

Talking itself could plunge them back into memories and emotional states that they preferred to forget.

*"So you see, I don't like to talk very much. If I talk and explain, it hurts. I start to cry..."* (interview 9)

Talking was therefore utilitarian and most often strategically oriented towards seeking protection. Some women explained that they had only spoken about the SV in order to tell the French Office for Refugees and Stateless Protection (OFPRA) or to ask for accommodation from an association.

In this way, the women expressed a sense of uselessness in speaking out, which tended to expand the silence surrounding the violence.

*“You're alone with your problems there. Even if you have a friend, you're going to tell her, and she's going to go and explain to someone else. You can't do it. You go through it alone; you live it alone.”* (Interview 15)

The women were able to speak out in spaces of trust and empathy with specific interlocutors who explicitly asked to hear their stories. The first prerequisite for speaking out was an active request from the other person and a genuine interest in them and their story.

*“If I'm asked, I speak. I'm looking for a solution, so I talk. When I'm asked, I talk about this.”* (interview 11)

General practitioners (GPs) were not identified as appropriate resources to talk to about SV.

*“The doctor, no, I don't talk about my stories. The illness, the results, yes, but I never discussed it with him. I talked to the other assistant. When I go there, he's a doctor, so in terms of health, I've never dared talk about it. These are life problems. I don't talk to him about life problems. With my social worker, I can talk to him about what I'm going through.”* (interview 15)

GPs could become a resource if a climate of trust was established and the question was asked, but they were primarily called upon to respond to complaints and requests associated with physical health.

### A contingent use of care in cases of SV, but with a very beneficial impact

Expectations in terms of care were initially low. The consequences identified as a result of SV were primarily psychological, including flashbacks, insomnia, fear, difficulties trusting others and a loss of self-confidence. The physical consequences the women perceived were mostly chronic pain.

“*Yes, I have problems with my head. Because sometimes... my head hurts every day!*” (Interview 16)

The first attitude described after SV was withdrawal based on the belief that the problems would resolve themselves naturally once their social situation was stabilized. The priorities for women were then having social rights and residence permit which could become a condition to access health care.

*“The fear of being, I don't know, expelled. And the fact that I don't know where to sleep, especially since I'm with the child. All that, all those factors. And also for health... for care! I don't know when the paper's going to run out, what am I going to do? In terms of the child, his injections, vaccinations and all that.”* (Interview 10)

The first use of care for the women was mostly to relieve physical complaints and the search for an aetiology were envisaged when problems became incapacitating. The women rarely explicitly requested care for SV, and their requests were dependent on referral by a third party (an association, social worker or family member). The psychological consequences of violence were dealt with during a discussion about the violence when the third party suggested psychological care, which had not been envisaged at the start of the meeting. The social worker played a central role in women's care after SV: they could refer them to health professionals or facilitate contact with the police. Time and repeated encounters favoured trust and discussion about SV, its consequences and possibilities for care.

“*It was my social worker who told me that "if you want gynaecologists", because sometimes I tell her that I'm in pain and all […] Then, my social worker called the police. When the police came, they said "our number is here, when the gentleman comes back you must call us right away." And he hasn't come back since*.” (Interview 16)

The more isolated and precarious the women were, the less likely they were to be referred for care. When such care was available, it was perceived as highly beneficial, with patients expressing a reduction in their symptoms and relief through talking and medication. This relative relief enabled them to be reintegrated into society.

"*Before, I didn't go out, I was locked up all the time. I was scared. Now I'm fine. I'm just waiting for my residence permit*”. (interview 2)

The women also reported receiving care through social events, such as French lessons, outings and social events organized by associations. Many said they were very isolated and had no strong friendships or emotional ties in France. The social events helped to decrease their isolation and represented a manner of care for the women. These events provided an opportunity to meet other women with similar problems and to share moments of joy in safe spaces.

"*With the association, above all, they go out. We go there. We have fun. I go to French lessons. When you go to French lessons, you have a laugh together. You talk. It's good enough”*. (interview 2)

### A representation of seeking care in cases of SV, shaped by past experiences of care

Dealing with SV meant discovering possibilities for care. It triggered a process of destigmatization of SV and its associated care. The women expressed their reluctance to receive psychological care at first, as it was culturally associated with weakness and madness. The support by a trusted third party, the experience of the care itself and the usefulness of care normalized it.

"*Back home, they say that if you go to see a doctor, it's because you're not right in the head. That's how it is. This is where they said, where I learnt, that psychologists help you to feel better, to free yourself if you've been through hard things and all that. He's someone I can talk to. You can... there you go*." (interview 10)

The connotation of speech itself changed from painful and dangerous to a sense of being heard safely and experiencing relief.

"*The fact of talking about it in general terms and understanding... trying to understand women who have experienced violence. It was new for me, because I didn't know that... that there were any procedures in place to deal with it*". (Interview 14)

"*I hope she'll make me say what's on my mind. Because I've got lots of things I can't say.”* (interview 1)

The way they looked at medication was also changing, with a reluctance to take it changing as they experimented with therapies, and this was accompanied by a improvement in their well-being.

“*That fear I had when I was walking down the street. Fear of being assaulted… the lack of confidence... I had in myself. That's starting to solidify a bit because it's still recent. They're trying to give me this so I can get on with my life and forget the past. I mean, I can't forget it! I can't forget it! Life goes on, life is beautiful. There are other opportunities waiting for me*.” (Interview 14)

The impact of SV on the women's health, which was often reduced to visible physical indicators such as scars, became more global, with a link made to all physical and psychological consequences. The discovery that sexual mutilation can be repaired by surgery, for example, has changed the perception of an irremediable and sometimes trivialized act.

"*I've also been mutilated. I have an appointment at the hospital on Monday. I can't wait for them to explain. I've been waiting 4 months. I don't really know what they can do*" (interview 3).

### Grounded theory

Our findings led us to develop the grounded theory shown in Fig. 1, which sets out the conditions for the recovery of ASW who have been victims of SV. These women are vulnerable to SV (social isolation, unstable housing and legal status), which re-exposes them to this violence and hinders their access to care in the host country. Initially motivated by a request for protection or by physical health needs, the disclosure of SV to a healthcare professional can lead to care that responds to the specific problems encountered by these women. To begin with, this presupposes a pro-active attitude on the part of healthcare professionals towards SV and the establishment of a climate of trust based on their posture. Secondly, the scope of care should be extended to include mental health and holistic, long-term and easily accessible care to these women. Initially trivialised, SV becomes a traumatic event from which they must be protected and for which they can benefit from care in order to envisage health recovery.

## Discussion

Preventing SV from becoming the norm among women who have experienced it many times in their lives requires disclosure. Experiences of care leading to concrete solutions to the problems encountered by victims (administrative stabilization, sheltering in a safe place, mental health care, appropriate treatment) strengthen victims' ability to disclose the SV they have experienced. This need for revelation and the link with the identified solutions has been reported in previous studies (De Schrijver et al., 2022; Azadi et al., 2021).

This qualitative section of the INCIDAVI study gives us a better understanding of the lack of access to care and the insecurity of these women, reported in our quantitative section (Khouani et al., 2023). The failure of ASW to seek care for SV is shaped by the fact that SV is part of their lives and is ordinary. However, a proactive attitude on the part of carers towards detecting such violence leads to positive experiences of care, which in turn influence women's initial perceptions of SV, enabling them to envisage health recovery. Furthermore, the significant association found in our quantitative phase between the lack of support for accommodation and sexual assault is clarified by our results. The instability of their accommodation led women to seek emergency solutions that could expose them to danger, for example, by being attacked by individuals who housed them or when they went to squats. Vulnerability to housing-related sexual violence is combined with social isolation and unstable administrative status. These have already been described in previous studies and appear to be common vulnerabilities for all female migrants who have recently arrived in a host country (Ouanhnon et al., 2023).

Future studies could focus on developing and evaluating protocols for screening for SV and caring for women who have experienced SV in this specific population. These protocols should account for the cultural, administrative and social barriers that these populations may face using a multidisciplinary approach that considers intercultural and health mediation. Given the importance attached to primary care by the WHO for the performance of health systems (World Health Organization, 2008) and the objective of universal health coverage, (https://www.who.int/fr/news-room/fact-sheets/detail/universal-health-coverage-(uhc)) these protocols could be studied by primary care services providing early care for ASW as close as possible to where they live. Particular attention will need to be focused on sexual and reproductive health among these women (Calderón-Jaramillo et al., 2020; Rivillas-García et al., 2021). Other experimental studies could compare the impact of stabilized administrative conditions (residence permits) and accommodation on the occurrence of SV in host countries or on the care of women experiencing SV that occurred prior to arrival. Now that the economic consequences of SV have been described, (Borumandnia et al., 2020) an assessment of the medico-economic efficiency of such a reception policy could be studied.

One of the limitations of our study is that we did not study the mechanisms by which male asylum seekers or minors, who are also vulnerable to SV, (Lay and Papadopoulos, 2009) seek care. Our sample consisted mainly of French speaking women from West Africa. This limitation is explained by the fact that our quantitative phase found a significant association between this geographical origin and rape or attempted rape. We therefore wanted to sample according to this population in particular. Also, fluency in French or English simplified telephone contact. Finally, it would have been relevant to study the care experiences and feelings of practitioners in the care of ASW who have been victims of SV to study the barriers to detecting violence.

## Conclusion

The failure of ASW to seek care for SV is shaped the fact that SV is initially perceived as ordinary. A proactive attitude and appropriate care on the part of carers towards detecting such violence leads to positive experiences, which in turn influence women's initial perceptions of SV, enabling them to envisage health recovery.

## Research in context

### Evidence before this study

Previous European studies have investigated SV suffered by ASW. Reducing social isolation, improving sexual health education and providing a space for victims to report their experiences have been described as measures that could help better prevent and manage care for women who have suffered SV among this population. All these studies highlighted the need to improve knowledge in this area. More recently, we led the INCIDAVI project (Incidence of sexual violence among recently arrived asylum-seeking women in France). Its quantitative phase found a 75 % prevalence of SV before arriving in the host country and a 26 % incidence of SV after arrival in the course of the year. More specifically, when violence occurs in the host country, more than one in two women do not seek any help and less than one in 10 seek medical help. Exploring the experiences of ASW suffering SV would give us a better understanding of how we can address unmet health needs and lack of social support.

### Added value of this study

We found that sexual violence is so frequent throughout their lives that it is initially trivialised by ASW. If this trivialisation is broken through screening and holistic care, ASW will be able to consider health recovery.

### Implications of all the available evidence

Despite its limitations, our study enables us to understand why female asylum seekers do not seek care when sexual violence occurs in the host country. It helps us to identify the right attitude to adopt when considering with such violence, and to identify strategies for care. Finally, it highlights the absolute need for legal and social interventions to prevent such violence in host countries.

## Funding

DGOS-GIRCI.

## Data sharing statement

Deidentified participant collected data, including individual participant data, will be made available by the corresponding author upon reasonable request.

## CRediT authorship contribution statement

**Khouani Jeremy:** Writing – review & editing, Writing – original draft, Validation, Methodology, Investigation, Funding acquisition, Conceptualization. **Anne Desrues:** Writing – review & editing, Investigation, Formal analysis, Data curation. **Constance Decloitre-Amiard:** Formal analysis, Data curation. **Marion Landrin:** Methodology, Funding acquisition, Data curation. **Rachel Cohen Boulakia:** Methodology, Funding acquisition, Data curation. **Didier Thery:** Writing – review & editing, Supervision. **Gaëtan Gentile:** Writing – review & editing, Supervision. **Pascal Auquier:** Writing – review & editing, Validation, Supervision, Funding acquisition, Conceptualization. **Maeva Jego:** Writing – review & editing, Writing – original draft, Validation, Supervision, Methodology, Formal analysis, Conceptualization.

## Declaration of competing interest

The authors declare that they have no known competing financial interests or personal relationships that could have appeared to influence the work reported in this paper.

## Appendix Supplementary materials

Image, application 1

Image, application 2

Image, application 3

Image, application 4

## References

[bib0025] Azadi B., Tantet C., Sylla F., Andro A. (2021). Women who have undergone female genital mutilation/cutting's perceptions and experiences with healthcare providers in Paris. Cult. Health Sex..

[bib0031] Borumandnia N, Khadembashi N, Tabatabaei M, Alavi Majd H. (2020). The prevalence rate of sexual violence worldwide: a trend analysis. BMC Public Health.

[bib0029] Calderón-Jaramillo M, Mendoza Á, Acevedo N, Forero-Martínez LJ, Sánchez SM, Rivillas-García JC. (2020). How to adapt sexual and reproductive health services to the needs and circumstances of trans people— a qualitative study in Colombia. Int. J. Equity Health.

[bib0009] Cayreyre L, Korchia T, Loundou A (2024). Lifetime sexual violence experienced by women asylum seekers and refugees hosted in high-income countries: literature review and meta-analysis. J. Forensic Leg. Med..

[bib0005] Cignacco E, Zu Sayn-Wittgenstein F, Sénac C (2018). Sexual and reproductive healthcare for women asylum seekers in Switzerland: a multi-method evaluation. BMC Health Serv. Res..

[bib0015] De Schrijver L, Nobels A, Harb J (2022). Victimization of applicants for international protection residing in belgium: sexual violence and help-seeking behavior. Int. J. Environ. Res. Public Health.

[bib0007] European Union (Eurostat) Annual asylum statistics [dataset]. https://ec.europa.eu/eurostat/statistics-explained/index.php?title=Annual_asylum_statistics.

[bib0023] Glaser BG, Strauss AL. (2009).

[bib0017] Keygnaert I, Guieu A. (2015). What the eye does not see: a critical interpretive synthesis of European union policies addressing sexual violence in vulnerable migrants. Reprod. Health Matters.

[bib0016] Keygnaert I, Vettenburg N, Temmerman M. (2012). Hidden violence is silent rape: sexual and gender-based violence in refugees, asylum seekers and undocumented migrants in Belgium and the Netherlands. Cult. Health Sex..

[bib0014] Khouani J, Landrin M, Boulakia RC (2023). Incidence of sexual violence among recently arrived asylum-seeking women in France: a retrospective cohort study. Lancet Reg. Health Eur..

[bib0032] Lay M, Papadopoulos I. (2009). Sexual maltreatment of unaccompanied asylum-seeking minors from the horn of Africa: a mixed method study focusing on vulnerability and prevention. Child Abuse Negl..

[bib0022] Lebeau J-P, Aubin-Auger I, Cadwallader J-S (2021).

[bib0021] Lejeune C. (2014). Analyser Sans Compter ni Classer.

[bib0006] Marek E, D'Cruz G, Katz Z, Szilard I, Berenyi K, Feiszt Z (2019). Improving asylum seekers' health awareness in a hungarian refugee reception centre. Health Promot. Int..

[bib0020] Marshall MN. (1996). Sampling for qualitative research. Fam. Pract..

[bib0011] Masterson AR, Usta J, Gupta J, Ettinger AS. (2014). Assessment of reproductive health and violence against women among displaced Syrians in Lebanon. BMC Womens Health.

[bib0010] Nesterko Y, Schönenberg K, Glaesmer H. (2021). Sexual violence and mental health in male and female refugees newly arrived in Germany. Dtsch. Arztebl. Int..

[bib0008] Office Français de Protection Des Refugiés Et Des Apatrides (OFPRA). Rapport d’activité. https://www.ofpra.gouv.fr/publications/les-rapports-dactivite, 2022 (accessed 28 July 2024).

[bib0018] Oliveira C, Keygnaert I, Oliveira Martins MDR, Dias S (2018). Assessing reported cases of sexual and gender-based violence, causes and preventive strategies, in European asylum reception facilities. Glob. Health.

[bib0026] Ouanhnon L, Astruc P, Freyens A, Mesthé P, Pariente K, Rougé D, Gimenez L, Rougé-Bugat ME (2023). Women's health in migrant populations: a qualitative study in France. Eur. J. Public Health.

[bib0024] Paillé P, Mucchielli A. (2021).

[bib0019] Palinkas LA, Horwitz SM, Green CA, Wisdom JP, Duan N, Hoagwood K. (2015). Purposeful sampling for qualitative data collection and analysis in mixed method implementation research. Adm. Policy Ment. Health.

[bib0030] Rivillas-García JC, Cifuentes-Avellaneda Á, Ariza-Abril JS, Sánchez-Molano M, Rivera-Montero D. (2021). Venezuelan migrants and access to contraception in Colombia: A mixed research approach towards understanding patterns of inequality. J. Migr. Health.

[bib0002] Sardinha L, Maheu-Giroux M, Stöckl H, Meyer SR, García-Moreno C. (2022). Global, regional, and national prevalence estimates of physical or sexual, or both, intimate partner violence against women in 2018. Lancet.

[bib0012] United Nations High Commissioner for Refugees (UNHCR). Sexual violence against refugees: guidelines on prevention and response. https://www.unhcr.org/publications/operations/3b9cc26c4/sexual-violence-against-refugees-guidelines-prevention-response-unhcr.html. 1995 (accessed 28 July 2024).

[bib0013] United Nations High Commissioner for Refugees (UNHCR). La violence sexuelle et sexiste contre les réfugiés, les rapatriés et les personnes déplacées. Principes directeurs pour la prévention et l’intervention. https://www.unhcr.org/fr-fr/publications/operations/4ad2f840e/violence-sexuelle-sexiste-contre-refugies-rapatries-personnes-deplacees.html.

[bib0003] World Health Organisation (WHO). Global, regional and national estimates for intimate partner violence against females and global and regional estimates for non-partner sexual violence against females. https://www.who.int/publications/i/item/9789240022256. 2021 (accessed 28 July 2024).

[bib0004] World Health Organisation (WHO). The health of refugees and migrants in the WHO European region. https://www.euro.who.int/fr/publications/html/report-on-the-health-of-refugees-and-migrants-in-the-who-european-region-no-public-health-without-refugee-and-migrant-health-2018/en/index.html, 2018 (accessed 28 July 2024).

[bib0001] World Health Organization (WHO) (2017).

[bib0027] World Health Organization (2008).

[bib0028] World Health Organization Universal health coverage - key fact. https://www.who.int/fr/news-room/fact-sheets/detail/universal-health-coverage-(uhc).

