# Supplementary material for: Strategies for seeking care in the host country among asylum-seeking women who have been victims of sexual violence: A French qualitative study
Source: J Migr Health. 2024 Jul 27;10:100254. doi: 10.1016/j.jmh.2024.100254 (PMC11341964; doi:10.1016/j.jmh.2024.100254)
Supplement: Supplementary file 1 [file mmc1.docx]

Supplementary material - COREQ checklist

| Domain 1 : research team and reflexivity |  |  |
| --- | --- | --- |
| 1. Interviewer/facilitator | Which author conducted the interview or focus group ? | DECLOITRE-AMIARD Constance and DESRUES Anne conducted the interviews.  P7 |
| 1. Credentials | What were the researcher’s credentials ? | DECLOITRE-AMIARD Constance is an MD.  DESRUES Anne has a Master’s in sociology and was working as investigator for Aix-Marseille University in the quantitative phase of the INCIDAVI Project  This work was codirected by Dr JEGO Maeva (MD-PHD, senior registrar at Aix-Marseille University)  and Dr KHOUANI Jeremy (MD, PHD student, general practitioner, senior registrar at Aix-Marseille University).  Title Page |
| 1. Occupation | What was their occupation at the time of the study ? | DECLOITRE-AMIARD Constance: MD student, resident in general practice.  DESRUES Anne : Working as interviewer for the quantitative phase of the INCIDAVI study.  JEGO Maeva: general practitioner (multiprofessionnal Health Center « Peyssonnel », 13003, Marseille), senior registrar at Aix-Marseille University (department of general practice).  KHOUANI Jeremy: general practitioner (multiprofessionnal Health Center « Peyssonnel », 13003, Marseille), senior registrar at Aix-Marseille University (department of general practice).  Title page |
| 1. Gender | Was the researcher male or female ? | DECLOITRE-AMIARD Constance: female  DESRUES Anne : female  JEGO Maeva : female  KHOUANI Jérémy : male |
| 1. Experience and training relationship with participants | What experience or training did the researcher have ? | DECLOITRE-AMIARD Constance: read qualitative methods books before beginning the research. She received personnel training by the codirector of this research (JEGO Maeva).  DESRUES Anne : had worked on several qualitative sociological studies as an independent researcher and as a member of a public policy consulting firm. Her master’s thesis was also a qualitative study on gender role.  JEGO Maeva had performed qualitative studies for her master’s thesis and MD thesis. She directed 17 qualitative research studies for MD student in general practice. She is member of the GROUM-F (Groupe Universitaire de Recherche Qualitative Médicale Francophone) and participated in 4 workshops about qualitative research. She is expert for the CNGE (collège national des généralistes enseignants) for trainings in qualitative research. |
| 1. Relationship established | Was a relationship established prior to study commencement ? | Yes. |
| 1. Participant knowledge of the interviewer | What did the participants know about the researcher ? | The participants knew the interviewer' education diploma and background and were briefly explained the qualitative methodology purpose |
| 1. Interviewer characteristics | What characteristics were reported about the interviewer | D.C., resident in general practice, and A.D., sociologist |
| Domain 2 : study design |  |  |
| 1. Methodological orientation and theory | What methodological orientation was stated to underpin the study ? | Grounded theory approach  P6 |
| 1. Sampling | How were participant selected ? | Purposive method (purposeful variation sampling)  P7 |
| 1. Method of approach | How were participants approached ? | Interviews were conducted after the quantitative phase of our project, and participants in the initial cohort were specifically contacted to participate in this qualitative phase. Two trained English- and French-speaking researchers conducted face-to-face semistructured interviews at a partner hospital in a private place or by phone when participants did not wish to travel. The interviews were audio-recorded and then fully transcribed. A professional telephone interpretation service was used for non-French-speaking and non-English-speaking participants.  P7 |
| 1. Sample size | How many participants were in the study ? | 20 |
| 1. Nonparticipation setting | How many people refused to participate or dropped out ? | **Not interviewed :**  -Unreachable after 3 attempts (n=9)  -Did not wish to participate to the qualitative phase (n=7)  -Agreed to participate but missed the interview appointment (n=3)  Flow chart Figure 1 |
| 1. Setting of data collection | Where was the data collected ? | Mostly in an office at the main Service Hospital Partner, or by phone.  P7 |
| 1. Presence of nonparticipants | Was anyone else present besides the participants and researchers ? | A professional telephone interpreting service was used for non-French-speaking and non-English-speaking participants. These professional interpreters were experienced in interviewing asylum-seeking women |
| 1. Description of sample data collection | What are the important characteristics of the sample ? | Twenty women were interviewed between February 1^st^, 2022, and July 29^th^, 2022. Data saturation was reached at the 18th interview and confirmed by 19^th^ and 20^th^ interviews. The average duration of the interviews was 1 hour (from 15 minutes to 75 minutes). Most of the females (13 of 20) were from West Africa. None of them were from South America. All the females included had been victims of SV in France and/or before arriving in France. Of these, 4 had been victims of rape in France, 14 of other types of SV in France, and 17 of SV before arriving in France. Four of them had been victims of all these types of violence |
| 1. Interview guide | Were questions, prompts, guides provided by the authors ? Was it pilot tested ? | The questions were prompts.  The guide is provided in Supplementary material.  The guide was pilot tested (2 pilot test) |
| 1. Repeat interviews | Were repeat interviews carried out ? | No |
| 1. Audio/visual recording | Did the research use audio or visual recording to collect the data ? | Yes (audio recording)  P7 |
| 1. Field notes | Were field notes made during and/or after the interview or focus group ? | Yes  P8 |
| 1. Duration | What was the duration of the interviews or focus group ? | 60 minutes  P9 |
| 1. Data saturation | Was data saturation discussed ? | Yes  P9 |
| 1. Transcripts returned | Were transcripts returned to participants for comment and/or correction ? | No (due to the protocol and confidentiality policy). |
| Domain 3 : analysis and findings |  |  |
| 1. Number of data coders | How many data coders coded the data ? | 2 (DECLOITRE-AMIARD Constance and DESRUES Anne) supervised by JEGO Maeva. |
| 1. Description of coding tree | Did authors provide a description of the coding tree ? | Yes, see supplementary material |
| 1. Derivation of themes | Were themes identified in advance or derived from the data ? | Derived from the data |
| 1. Software | What software, if applicable, was used to manage the data ? | NVivo® release 14 software |
| 1. Participant checking reporting | Did participants provide feedback on the findings | No |
| 1. Quotations presented | Were participants quotations presented to illustrate the themes/findings ? Was each quotation identified ? | Yes.  Yes |
| 1. Data and findings consistent | Was there consistency between the data presented and the findings ? | Yes |
| 1. Clarity of major themes | Were major themes clearly presented in the findings ? | Yes  Results section |
| 1. Clarity of minor themes | Is there a description of diverse cases or discussion of minor themes ? | Yes |
