# Supplementary material for: Strategies for seeking care in the host country among asylum-seeking women who have been victims of sexual violence: A French qualitative study
Source: J Migr Health. 2024 Jul 27;10:100254. doi: 10.1016/j.jmh.2024.100254 (PMC11341964; doi:10.1016/j.jmh.2024.100254)
Supplement: Supplementary file 2 [file mmc2.pdf]

Editing Certificate

This document certifies that the manuscript

Strategies for seeking care in the host country among asylum-seeking women who have been victims of sexual violence: A French qualitative study

prepared by the authors

Khouani Jeremy, Desrues Anne, Constance Decloitre-Amiard, Marion Landrin, Rachel Cohen Boulakia, Jenny Forte, Didier Thery, Gaëtan Gentile, Auquier Pascal, Jego Maeva,

was edited for proper English language, grammar, punctuation, spelling, and overall style by one or more of the highly qualified native English speaking editors at AJE.

This certificate was issued on **November 13, 2023** and may be verified on the AJE website using the verification code **9221-ADDD-7686-9FC5-D52P**.

Neither the research content nor the authors' intentions were altered in any way during the editing process. Documents receiving this certification should be English-ready for publication; however, the author has the ability to accept or reject our suggestions and changes. To verify the final AJE edited version, please visit our verification page at aje.com/certificate. If you have any questions or concerns about this edited document, please contact AJE at support@aje.com.

AJE provides a range of editing, translation, and manuscript services for researchers and publishers around the world. For more information about our company, services, and partner discounts, please visit aje.com.
