# Supplementary material for: Strategies for seeking care in the host country among asylum-seeking women who have been victims of sexual violence: A French qualitative study
Source: J Migr Health. 2024 Jul 27;10:100254. doi: 10.1016/j.jmh.2024.100254 (PMC11341964; doi:10.1016/j.jmh.2024.100254)
Supplement: Supplementary file 3 [file mmc3.pdf]

INCIDAVI

Semi-structured interview guide

1/

How were the care you received following the violence you suffered in France ?

Prompt : What was your pathway to care? Which carers did you go to see?

1bis/

If the patient did not seek care

Why didn't you confide in a carer?

Prompt : Did you try to contact a health facility (hospital, emergency, general practitioner, etc.)? What would it have taken for you to consult a doctor?

2/

How do you feel about the French healthcare system?

Prompt: Tell me about a healthcare experience you have had in France.

3/

What difficulties did you encounter when you wanted to consult a doctor?

4/

What could have made it easier for you to consult a doctor or other health care provider? How could it have been easier for you?
