# Supplementary material for: Strategies for seeking care in the host country among asylum-seeking women who have been victims of sexual violence: A French qualitative study
Source: J Migr Health. 2024 Jul 27;10:100254. doi: 10.1016/j.jmh.2024.100254 (PMC11341964; doi:10.1016/j.jmh.2024.100254)
Supplement: Supplementary file 4 [file mmc4.pptx]

## Slide 1
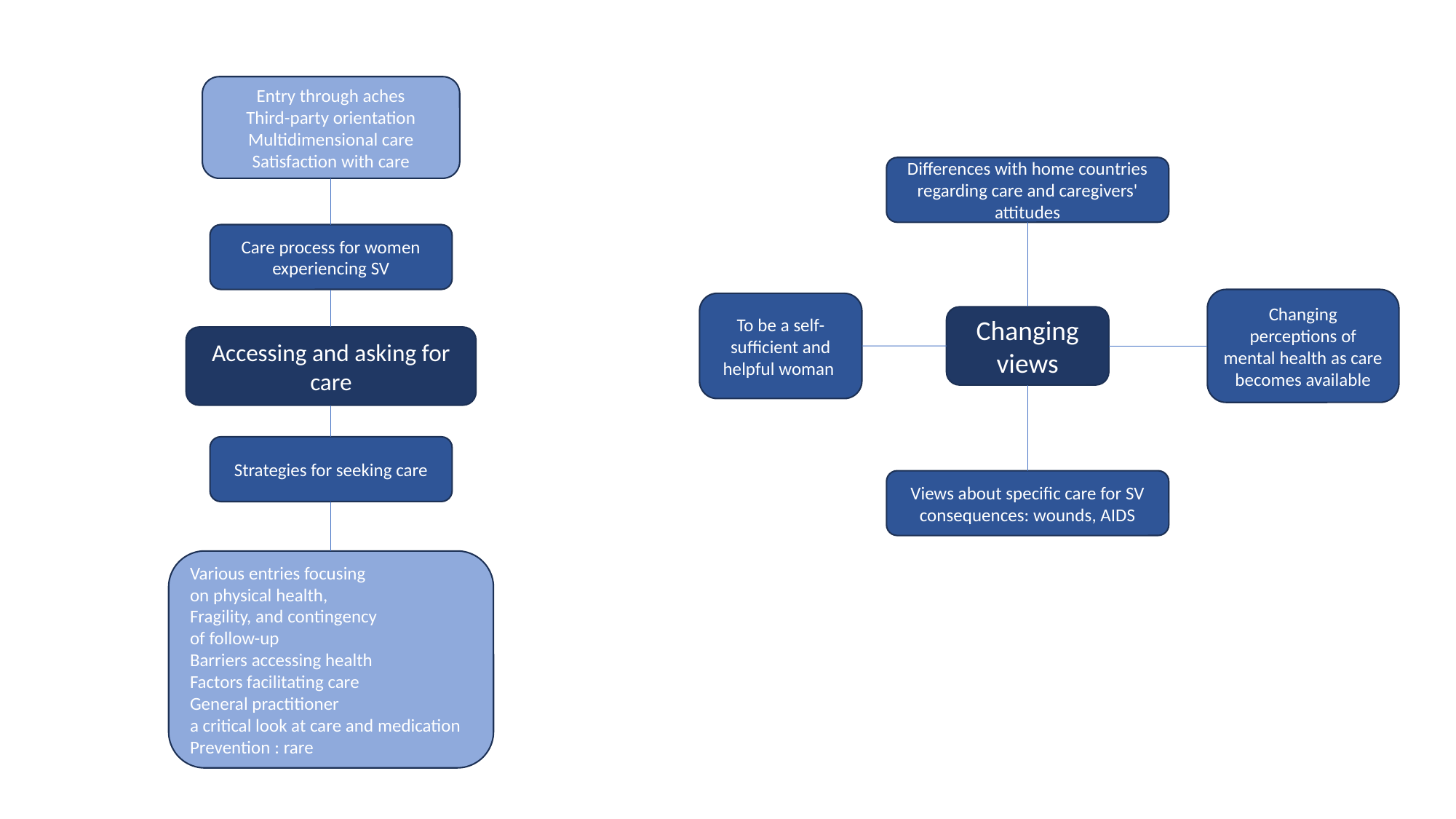

Entry through aches
Third-party orientation
Multidimensional care
Satisfaction with care
Differences with home countries regarding care and caregivers' attitudes
Care process for women experiencing SV
Changing perceptions of mental health as care becomes available
To be a self-sufficient and helpful woman
Changing views
Accessing and asking for care
Strategies for seeking care
Views about specific care for SV consequences: wounds, AIDS
Various entries focusing
on physical health,
Fragility, and contingency
of follow-up
Barriers accessing health
Factors facilitating care
General practitioner
a critical look at care and medication
Prevention : rare

## Slide 2
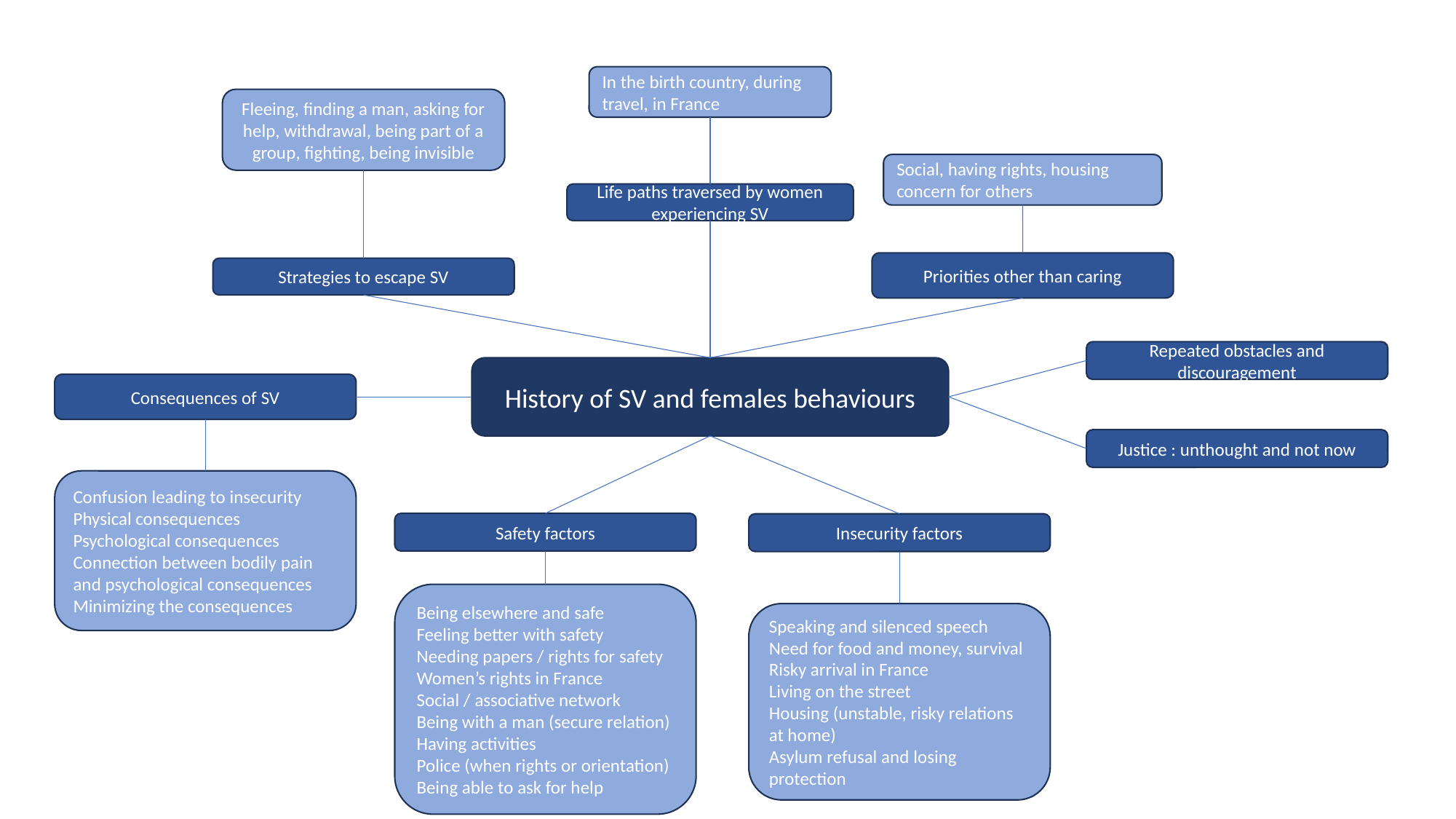

In the birth country, during travel, in France
Fleeing, finding a man, asking for help, withdrawal, being part of a group, fighting, being invisible
Social, having rights, housing
concern for others
Life paths traversed by women experiencing SV
Priorities other than caring
Strategies to escape SV
Repeated obstacles and discouragement
History of SV and females behaviours
Consequences of SV
Justice : unthought and not now
Confusion leading to insecurity
Physical consequences
Psychological consequences
Connection between bodily pain and psychological consequences
Minimizing the consequences
Safety factors
Insecurity factors
Being elsewhere and safe
Feeling better with safety
Needing papers / rights for safety
Women’s rights in France
Social / associative network
Being with a man (secure relation)
Having activities
Police (when rights or orientation)
Being able to ask for help
Speaking and silenced speech
Need for food and money, survival
Risky arrival in France
Living on the street
Housing (unstable, risky relations at home)
Asylum refusal and losing protection

## Slide 3
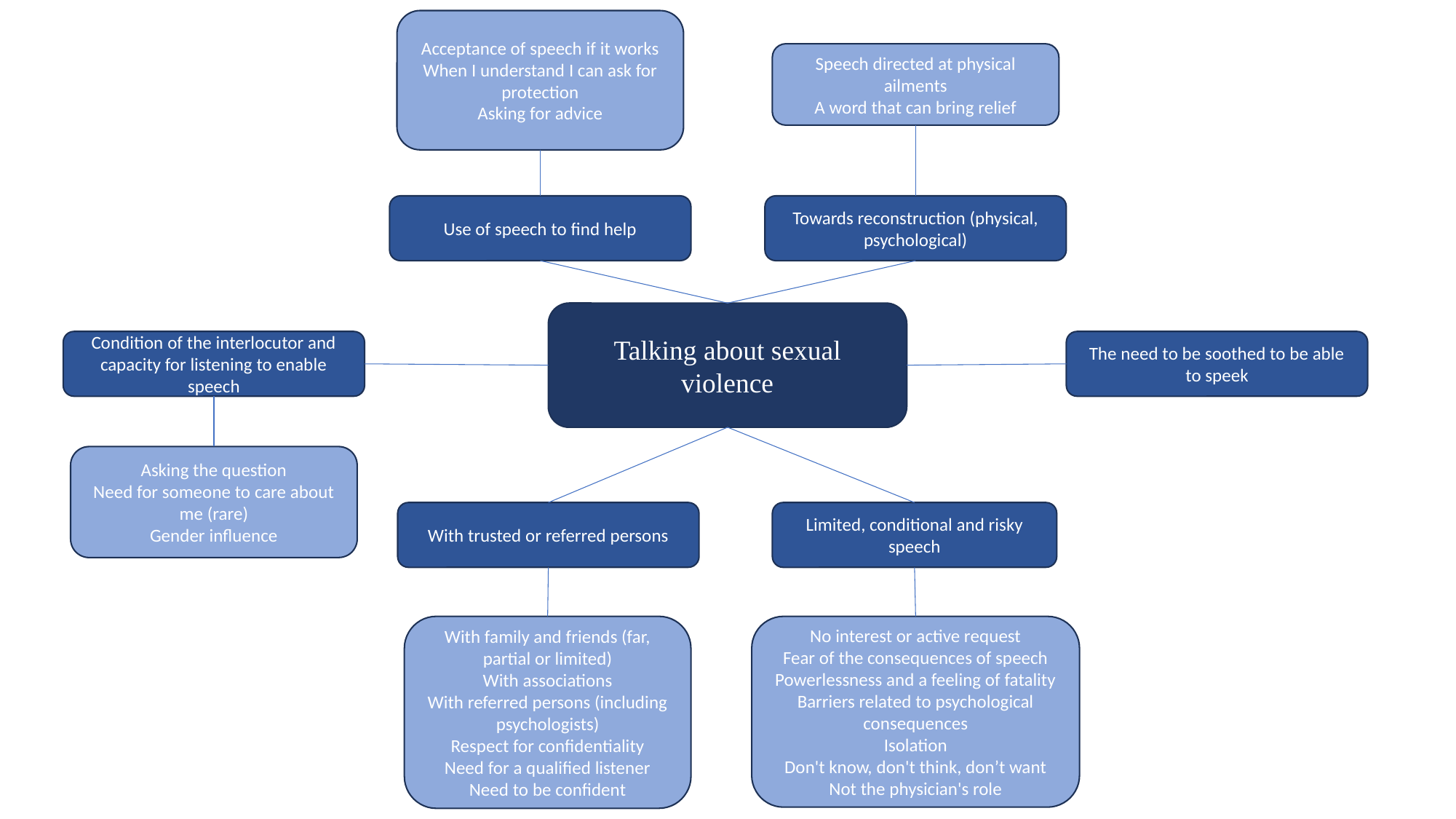

Acceptance of speech if it works
When I understand I can ask for protection
Asking for advice
Speech directed at physical ailments
A word that can bring relief
Towards reconstruction (physical, psychological)
Use of speech to find help
Talking about sexual violence
Condition of the interlocutor and capacity for listening to enable speech
The need to be soothed to be able to speek
Asking the question
Need for someone to care about me (rare)
Gender influence
With trusted or referred persons
Limited, conditional and risky speech
With family and friends (far, partial or limited)
With associations
With referred persons (including psychologists)
Respect for confidentiality
Need for a qualified listener
Need to be confident
No interest or active request
Fear of the consequences of speech
Powerlessness and a feeling of fatality
Barriers related to psychological consequences
Isolation
Don't know, don't think, don’t want
Not the physician's role
